# Interventions that prevent or reduce perinatal loneliness and its proximal determinants: a restricted scoping review

**DOI:** 10.1186/s12889-024-20788-z

**Published:** 2025-02-06

**Authors:** Ruth Naughton-Doe, Rebecca Nowland, Stephanie Tierney, Martin Webber, Anja Wittkowski

**Affiliations:** 1 University of York, York, UK; 2 University of Central Lancashire, Preston, UK; 3 University of Oxford, Oxford, UK; 4 The Perinatal Mental Health and Parenting (PRIME) Research Unit, Greater Manchester Mental Health NHS Foundation Trust, Manchester, UK

**Keywords:** Loneliness, Mothers, Fathers, Parents, Perinatal loneliness, Perinatal mental health, Interventions, Scoping review, Isolation; support

## Abstract

**Background:**

The World Health Organisation’s Commission on Social Connection (2024–2026) highlights the importance of addressing loneliness because of its negative impact on health and well-being. The perinatal period carries an increased risk of loneliness for mothers and fathers which is elevated by intersectional inequalities, such as having a low income, being LGBTQ+, or being from a minoritised community. Perinatal loneliness is associated with perinatal mental illness, which can have lasting negative impacts on parents and their children. The aim of this review was to synthesise studies exploring interventions for perinatal loneliness.

**Methods:**

We conducted a restricted scoping review following the Joanna Briggs Institute Methodology to develop a categorisation of interventions and intervention-mechanisms to reduce perinatal loneliness. We included studies that described and/or evaluated interventions in published studies that intentionally or unintentionally reduced loneliness, or its proximate determinants, such as social connectedness and social support. We searched eight electronic databases for peer-reviewed academic papers published in any country describing or evaluating these interventions between 2013–2023.

**Results:**

Fifty papers were included in the review, from which the following categorisation of interventions was developed: 1) synthetic social support, 2) shared-identity social support groups, 3) parent and baby groups, 4) creative health approaches (arts, nature or exercise based), 5) holistic, place-based and multidisciplinary support that worked with parents to overcome a range of barriers to connection, and 6) awareness campaigns. Five mechanisms were identified within included papers: 1) opportunities for social connection to similar others, 2) positive relationships with a professional or volunteer, 3) normalisation and acceptance of difficulties, 4) meaningful activities and 5) support to overcome barriers (including cultural and financial) to connection. Few studies collected comprehensive demographic data, few considered fathers, and none were LGBTQ+ specific.

**Conclusions:**

The review identified and synthesised approaches that might address perinatal loneliness and its proximate determinants. Further research is needed to scope the grey literature, review papers in the global south, appraise intervention effectiveness, and co-produce interventions, including for fathers, LGBTQ+ parents, and cultural and religious minorities.

**Trial registration:**

The protocol for the trial was registered on Figshare.

## Background

Loneliness can be experienced across the life course although it is more likely during major life transitions, such as becoming a parent [1]. Perinatal loneliness, which refers to loneliness experienced during pregnancy and up to two years post-birth, is linked to poor mental health, such as postnatal depression [2–6]. Most studies exploring perinatal loneliness have been conducted in high- and middle-income countries [7], despite the likelihood of loneliness also affecting parents in low-income countries [8]. The World Health Organisation Commission on Global Connection 2024–28 has identified reducing loneliness as a public health and policy priority due to its association with poor outcomes and reduced life expectancy [9]. Furthermore, tackling loneliness in new parents was identified as a priority in the 2022 UK Government Loneliness Strategy due to a dearth of evidence identifying solutions [10, 11]. In the United Kingdom (UK), perinatal mental illness is estimated to cost the government £8.1bn per year, with much of the costs attributed to lasting impacts on children [12].

Loneliness is commonly experienced when there is a gap between an individual’s desired and actual social networks. Weiss’s [13] dominant conceptualisation outlines two main experiences – social and emotional. Social loneliness refers to having limited social contacts, such as few friends, neighbours or colleagues. Emotional loneliness refers to the absence of a meaningful relationship or someone to confide in, such as a partner or close friend. A third dimension, existential loneliness, refers to feelings of meaningless, separateness and being misunderstood [14, 15]. Existential loneliness has been less explored, but recent studies with both younger [16] and older people [17] emphasised that it was worthy of more consideration. Existential loneliness may be particularly pertinent to perinatal experiences given that new parents struggle with dislocation of self and identity [18, 19].

Social support, social isolation and social connectivity are terms often conflated with loneliness but whilst related, describe different concepts. Loneliness is a subjective experience, whereas being socially isolated can be objectively measured by examining a person’s social connections [20]. Social support describes the availability and/or quality of support a person has [21] and social connectivity refers to the networks available to someone if they seek support [2]. Social support can either be spontaneous, from existing networks, or ‘synthetic’ [22], such as time-limited and non-reciprocal support offered by professionals or through formal peer support relationships. Recognising their distinctiveness as well as their relationship and association with loneliness, social support, social isolation and social connectivity have been described and understood as proximal determinants of loneliness [7, 23, 24].

Previous studies [2, 3] suggest universal causes of perinatal loneliness, including difficulties in adjusting to parenthood, and being cut off from social support. Other research reports that parents experienced a loss of identity and fear judgement if they shared their difficulties [7, 18, 25]. Parents are more at risk of loneliness if they are experiencing socioeconomic deprivation, physical or mental ill health, are living with a disability, are solo parents, or from certain minority ethnic backgrounds; intersectional inequalities also compound the risks [3–5].

Given the potentially profound and detrimental impacts of perinatal loneliness, it is important to identify effective interventions. Systematic or scoping literature reviews of interventions to reduce loneliness have so far focussed on older adults [26–28] or specific populations, such as university students [29], young people [30] or ethnic-minority groups [23]. Although there are no published reviews specifically exploring interventions to reduce perinatal loneliness, there are five published reviews with related or overlapping aims. These reviews are summarised in Table 1 and include: i) a meta-synthesis of 27 qualitative studies that explored the experience of loneliness in perinatal depression [7], ii) a realist synthesis of 27 studies synthesising evidence about social connectivity interventions during the transition to parenthood [2], iii) a systematic review of 68 studies that explored interventions for non-elderly populations, including six which were interventions for new parents [31], iv) a scoping review of 108 studies that explored the experiences of loneliness in pregnant and postpartum people of children aged 0–5 [3], and v) a scoping review of 133 studies that explored loneliness in parents of children aged 0–16 that included a summary of 14 intervention studies, nine of which focussed on interventions for new mothers [4]. Noticably, none of these reviews explored interventions specifically for fathers, LGBTQ+ parents, adolescent parents, refugees, or migrant parents.

**Table 1:** Summary of published reviews exploring perinatal loneliness

Citation	Title of paper	Search dates	Search terms	Databases	No. of studies	No. of perinatal intervention studies	Interventions explored	Inclusion criteria	Exclusion criteria	Extracted data
Adlington, et al. (2023) [7]	‘Just snap out of it’ – the experience of loneliness in women with perinatal depression: a Meta-synthesis of qualitative studies	Completed July 2021	Search terms included (i) perinatal population, (ii) mental health disorders, (iii) loneliness and (iv) qualitative research. Search terms were inclusive to include social isolation, social network, social support and social connection	Ovid MEDLINE®; PsycINFO; Embase; Web of Science	27	Many of the participants in studies were recruited from interventions but did not focus on the intervention itself	Peer support from healthcare professionals and peer support from other mothers who have experienced perinatal depression	Studies in English where > 50% of the result section concerned participants talking about subjective experiences of loneliness or closely related themes and > 50% participants had personally experienced perinatal depression. For full inclusion criteria see	Participants with comorbid substance misuse disorders, participants who had experienced perinatal loss, loss, studies evaluating an intervention, mixed methods studies	Experiences of perinatal loneliness were downloaded into Nvivo
Bennett et al. (2017) [2]	A realist synthesis of social connectivity interventions during transition to parenthood: The value of relationships	2012	Social support, social environment, social capital, peer support, support network, social connect AND infant, parents, father, mother, parenting, pregnant, prenatal care and postnatal care	Medline, CINAHL, SocAbs, PsychINFO and grey literature sources	27	27	Exercise and support, Family Resource Centres, community-based early intervention, Early Childhood Group Intervention, peer support, antenatal classes, online, discussion groups, email, websites, internet groups and online information	Intervention studies published in English in the perinatal period, that included social support and with the potential for universal, population-level reach	Studies related to highly targeted populations were excluded. Studies that involved home visiting were also excluded	Country, study design, summary of intervention
Bessaha et al. (2020) [31]	A systematic review of loneliness interventions among non-elderly adults	In and before 2015	A range of terms for loneliness and intervention	CINAHL, Pubmed, PsycINFO, Social Work Abstracts	68	6 studies focussed on parental loneliness	Telephone peer support, Child Development Program, group CBT for trauma, Telehealth	Adults aged 18–65 Intervention studies	Interventions not specifically aimed at reducing loneliness	Type of intervention, study design, sample size, age range of participants, loneliness measure, statistical significance of study findings
Kent-Marvick et al. (2022) [3]	Loneliness in pregnant and postpartum people and parents of children aged 5 years or younger: a scoping review	Pre February 2020 (pre COVID-19 pandemic)	loneliness, lonely, pregnancy, pregnant, parenting, and parents. Grey literature was searched via Google search by the first author	MEDLINE EMBASE SCOPUS Cochrane Library including CENTRAL CINAHL PsycINFO Global, and Web of Science	108	N/A was not the aim	N/A was not the aim	Pregnant people or parents with children aged 5 years. All types of publications addressing loneliness within the target population were included in the review process, including grey literature and dissertations	Loneliness studies published during the pandemic. Studies with no English-language translation available	Citation, country of origin, study aim(s), study design, sample, study results/main outcomes, types of loneliness identified, definition(s) of loneliness used, factors associated with and protective of loneliness, and prevalence data
Nowland et al. (2021) [4]	Experiencing loneliness in parenthood: a scoping review	May 2019 to February 2020 (pre Covid-19 Pandemic)	Mother*, maternal, parent*, father*, paternal AND Lonel* or ‘perceived social isolat*’	PsycINFO, Medline, CINAHL, Embase, Web of Science and Scopus	133	14 studies focussed on parents including 9 studies that focussed on new mothers	Peer support variants (telephone, peer-led, technology-delivered, home-based), home visiting, online discussion forums, Child Development Program, group CBT for trauma, Telehealth	Mothers, fathers, (biological or step- parents) of children 16 years and under and living in the family home. All research designs and exploring loneliness/social isolation. English studies only	Non-parental caregivers, parents with children over the age of 16 and/or not living in the family home or studies that examined loneliness in child only, or pregnancy and birth experiences	Year, country, design, loneliness measure, child’s age, findings relating to experiences, attitudes and opinions of loneliness, prevalence of loneliness, impacts of parental loneliness on parent or child’s health and wellbeing

Three of these reviews [2, 4, 31] identified a range of interventions that show promise in reducing loneliness and its proximal determinants (see Table 1). These interventions include home visiting, family support interventions, group-based Cognitive Behavioural Therapy (CBT), group activities, such as walking groups, or group educational approaches, such as child development courses. Interventions were delivered in person, on the telephone, online, or using digital technology, such as online forums (see Table 1) [2, 4, 31].

The other two reviews [3, 7], whilst not identifying interventions, made recommendations for approaches to reduce loneliness based on their synthesis of parents’ and professionals’ experiences (see Table 1). These reviews suggested that parents should be provided with opportunities to connect with others with shared experiences either by themselves or with support from their community or healthcare services [3, 7].

The current scoping review reported in this paper was conducted as the first part of a research project that aimed to identify and/or develop potential solutions for perinatal loneliness in collaboration with parents with lived experience and the professionals who supported them [19]. To inform subsequent qualitative research, which involved discussing approaches with parents and professionals, a scoping review helped to build on previous reviews by providing an updated and comprehensive report of the types of interventions available. We reasoned there were likely to be many more papers published in recent years due to the rising awareness that loneliness in the perinatal period is a public health issue, especially considering the ongoing worldwide COVID-19 pandemic, which caused additional loneliness through social distancing policies [32].

This scoping review aimed to provide an overview of published academic literature describing or evaluating interventions for perinatal loneliness. Its specific aims were to: i) develop a categorisation of existing interventions for perinatal loneliness and its proximate determinants, ii) identify common mechanisms that might reduce perinatal loneliness, and iii) identify gaps in the current research and make recommendations for the focus of future research.

## Methods

### Scoping review and search strategy

Recommendations for conducting scoping reviews from the Joanna Briggs Institute (JBI) [33] were followed. A restricted review (sometimes known as a rapid review) was conducted due to practical limitations of allocated research time and funding and the need to incorporate up-to-date knowledge into further project work [34]. We utilised the *Selecting Approaches for a Rapid Reviews Decision Tool (StaRR)* [35] when deciding how to restrict the review without compromising rigour. Restrictions were that one reviewer (RND) searched the literature, screened abstracts, and selected full papers. Searches were also restricted to the past ten years (2013–2023) to focus on contemporaneous papers more likely to be contextually relevant given the aims of our study to inform intervention development. The protocol was published on Figshare [36].

Our review also sought to address potential gaps in the search strategies used in previous reviews. Although Bennet et al. [2] explored interventions in the perinatal period, the search was focussed on interventions that facilitated social connections in the transition to parenthood, and the review did not include loneliness in the search terms [2]. Bennet et al. [2] also excluded studies that related to groups of parents with specific needs as the focus was on public health interventions transferable to the general population. Nowland et al. [4] identified interventions specifically to reduce loneliness and isolation in parents, but their search terms included only ‘loneliness’ and ‘isolation’ which may have missed other variants such as social capital or social connectivity. Considering that the aim of our research project was to identify promising approaches to reduce perinatal loneliness, a pragmatic broad inclusion criterion that included overlapping concepts and proximate determinants was developed. In addition to social support, social capital and social connectivity [7], we included other proximate determinants, such as building or maintaining friendships [37]. We also included outcomes relating to existential loneliness, including alienation, or reconnecting to a sense of identity and community [23].

A search strategy was developed by the research team with support from an information specialist, and from advisory groups formed of practitioners and people with lived experience of loneliness. The search terms and limits are shown in Table 2.

**Table 2:** Search terms and limits

	**Categories searched**	**Where searched**	**Search terms and Boolan operators**
1	Sample	Title, Abstract and Keywords	(mum* OR mom* OR mama* OR papa* OR dad* OR mother* OR father* OR parent* OR perinatal OR postpartum OR antenatal OR maternal OR paternal OR pregnant OR pregnanc* OR prenatal OR postnatal OR childbearing OR "antenatal" OR pre-natal OR "childbearing" OR peripartum OR peri-natal OR puerperium OR "surrogate mothers" OR "adoptive famil*")
2	Phenomena of interest	Title, Abstract and Keywords	(lonely OR loneliness OR isolation OR isolated OR "social capital" OR "social network" OR "social connect*" OR "social relationship*" OR "social disconnect" OR "social interact*" OR friend* OR alienat* OR identit*)
3	Intervention	Title, Abstract & Keywords	(interven* OR solution* OR prevent* OR support OR help OR service* OR therap* OR befriend* OR playgroup OR leisure OR psychosocial OR education OR psychoeducation OR "perinatal mental health")
4	Limiters	Title	("school age*" or "primary school" or "fetus" or "foetus" or "infant school" or ultrasound)
		In Journal	(biology OR chemistry OR engineer* OR ultrasound OR neurology* OR dentist* OR toxicity OR placenta OR blood or cell)

Nine electronic databases were searched: the Applied Social Sciences Citation Index (PROQUEST), CENTRAL (Cochrane), CINAHL Ultimate (EBSCO), Maternity and Infant Care (Ovid), MEDLINE (Ovid), SCOPUS, PsychINFO (Ovid), Science Citation Index (Web of Science) and the Social Sciences Citation Index (Web of Science). These specific databases were chosen for their interdisciplinary focus and the potential to identify interventions spanning nursing, midwifery, psychology, social work and social care. The review searched for studies published in any country between January 2013 and 23 October 2023 (date of the final search).

### Eligibility criteria

The population, concept, and context (PCC) approach was used to facilitate the development of eligibility criteria and to standardise the screening approach [38, 39]. To be included in the review, studies had to describe and/or evaluate an intervention to address perinatal loneliness. Intervention is defined in this review as any process that aimed to reduce loneliness and/or proximal determinants for new or expecting parents; self-help strategies or support given by health and social care professionals, or voluntary sector organisations were included. Any parents were included, including step, foster and adoptive parents.

All types of qualitative, quantitative and mixed-methods primary research study designs were included but only if the intervention a) was specifically designed to reduce loneliness, b) unintentionally impacted loneliness or c) addressed proximal determinants such as social support and connectivity [23]. As we were interested in promising interventions, we only included studies that reported positive outcomes of interventions, including through themes identified through qualitative data analysis, participants’ self-reported changes on retrospective surveys, or changes to outcome measures across time.

Articles were excluded if they were not published in English, did not demonstrate positive results, were not published in a peer reviewed journal, were a systematic review, or related to perinatal loss. As our study aim was not to synthesise outcome data, we did not utilise exclusion criteria related to methodological quality.

### Study selection

All references identified from the search were uploaded and screened using Covidence systematic review software [40]. The first author screened the abstracts against the eligibility criteria and screened eligible full text papers for inclusion in the review. Full text papers that met the eligibility criteria were downloaded for data extraction. The second author was consulted on any uncertainties and checked a random sample of 10% of retrieved full text papers. There was one disagreement that was resolved through discussion and then the first author re-reviewed the remaining papers in line with the new consensus.

### Data extraction and analysis

Data from each study were extracted into a table and included details of the intervention described and/or evaluated, country of origin, how it was designed, participant demographics, and intervention components (including number of sessions, who delivered it and where). A narrative approach was then used to synthesise the characteristics of interventions and develop a categorisation. Similar types of interventions (e.g., peer support or interventions with a creative component) were grouped together to form categories. Some of the categories had been identified by previous research exploring social interventions [22, 23], whereas others were developed following analysis of the intervention components. These categories were refined through discussion with the research team and advisory group and presented in another table.

We extracted data to develop a categorisation of intervention mechanisms, which refer here to specific processes to reduce loneliness or impact on proximate determinants created through an intervention. Some studies specifically aimed to identify ‘mechanisms’ [22, 41–44], and others made suggestions on why or how interventions created social outcomes. The first author used inductive thematic analysis to develop preliminary themes of mechanisms overtly described or tacitly suggested in the studies [45]. The identified mechanisms were discussed with and refined by members of the research team, in advisory group meetings, and with colleagues in a *Parental Loneliness Research Group.* Data about mechanisms were then tabulated and were checked by the second author who reviewed 10% of the papers. Following a discussion, no changes were made.

Data about study methods and a descriptive summary of key findings about the impact of the interventions on loneliness and its proximate determinants were then extracted and tabulated. In line with our restricted scoping review methodology [33, 34], we did not quality appraise the included studies, so these findings are presented uncritically and without analyses of effect sizes, risk of bias, or reliability. The studies were diverse and explored different interventions and utilised varied methodologies and outcome measures. Consequently, a meta-analysis to compare efficacy was not possible.

## Results

### Overview of included studies

**Fig. 1:**
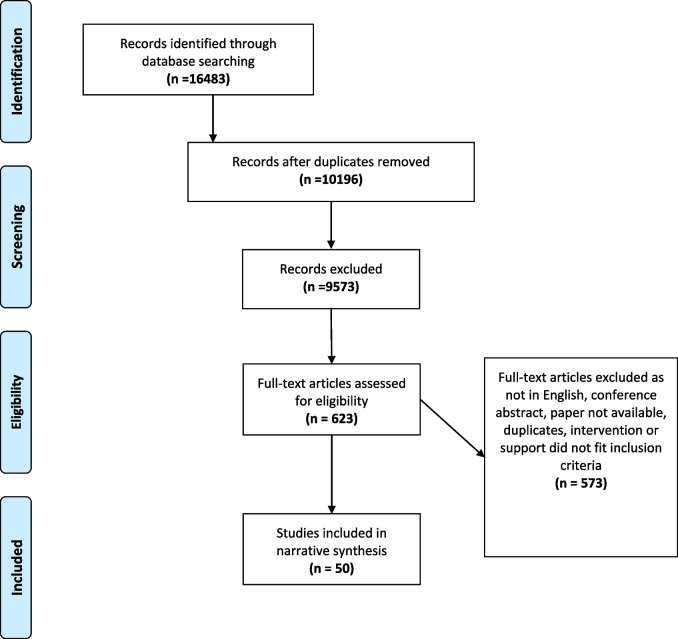
PRISMA flow diagram

Results of the screening process are presented in a PRISMA flow diagram in Fig. 1. After removing duplicates, 10,196 records were eligible for screening. Following title and abstract screening, 623 studies were retrieved for full review, of which 50 studies were considered eligible for inclusion (See Tables 3 & 4). The included studies described interventions conducted in the UK (*n* = 19), Australia (*n* = 10), United States of America (USA) (*n* = 7), Asia (*n* = 4), Scandinavia (*n* = 4), European countries (*n* = 3), Canada (*n* = 1), New Zealand (*n* = 1) and Turkey (*n* = 1).

**Table 3:** Intervention components

**Author/** **Date**	**Intervention overview**	**Who designed the intervention, and was it coproduced/co-designed?**	**Target population**	**Country **	**How it was delivered (e.g. groups/1-2-1)**	**Where was it delivered? (e.g. online or in person)**	**When was it delivered?**	**Length of intervention**
Anolak et al. (2003) [46]	Antenatal music drawing and narrative intervention	Research team was comprised of a midwife, music therapist, academics, and a medical student.	Pregnant mothers admitted to hospital due to complications	Australia	Group	In person	Antenatal	One session with up to two participants.
Aube et al. (2019) [47]	Wraparound holistic support for immigrants, refugees and asylum seekers	The community-based organisation who delivered the approach. Not codesigned or coproduced.	Migrant women	Canada	1-2-1 Drop-in Groups	In person	Perinatal	Continuous – for women who are pregnant or have children under 5.
Augustin et al. (2023) [48]	Psychoeducation App - support with early childhood crying, sleeping, and feeding problems	Not discussed but appears that the research team designed the intervention.	All parents	Germany	Self-help Drop-in Forum	Online	Postnatal	Mean use of app in the study was 10-49 days.
Berg et al. (2018) [49]	Web-based support, including peer support, for women with Type 1 Diabetes	Refers to participatory design but is unclear.	Type 1 Diabetic Mothers	Sweden	Forum Self-help	Online	Perinatal	No limit- pregnancy to early motherhood.
Bess et al. (2014) [50]	Place-based parent education initiative	A philanthropic Christian organisation. Not codesigned or coproduced.	Parents in local neighbourhood	USA	Group-based	In person	Perinatal	10-week programme with a range of activities.
Birtwell et al. (2013) [51]	Explore women’s experiences of Mellow Bumps intervention - an intervention to improve maternal well-being	Explored an established intervention – unclear whether coproduced or codesigned.	Vulnerable/ disadvantaged women	UK	Group	In person	Antenatal	Weekly sessions for 6 weeks.
Brookes et al. (2015) [52]	Antenatal classes- Baby Steps programme for minority ethnic parents	A University team and a Charity. Unclear if coproduced or codesigned.	Asylum seeking parents	UK	Facilitated Group sessions and 1-2-1s	In person	Perinatal	9 group-based sessions from 28 weeks pregnant.
Buston et al. (2019) [41]	Mellow Bumps intervention - an intervention to improve maternal well-being	Explored an established intervention. Unclear whether coproduced or codesigned.	Vulnerable/ disadvantaged women	UK	Facilitated Group sessions	In person	Antenatal	6 weekly sessions for two hours a week.
Buultjens et al. (2018) [53]	An antenatal 3^rd^ trimester psychoeducational group for first time parents	Developed by multidisciplinary team but unclear how or if coproduced.	First time mothers	Australia	Group -facilitation	In person	Perinatal	Weekly two-hour sessions 3^rd^ trimester and ending 8 weeks after birth.
Carter et al. (2020) [54]	Peer support intervention for antenatal depression	Unclear who designed the intervention.	Mothers at risk of depression	UK	1-2-1	In person	Antenatal	Weekly sessions for 6 weeks.
Chatwin et al. (2021) [55]	Online midwife facilitated virtual community on social media	National Health Service but unclear if coproduced or codesigned. It is being piloted.	Any pregnant woman	UK	Forums	Online	Perinatal	No limit - pregnancy and beyond
Darra et al. (2020) [56]	Multi-agency project to support young parents	Developed through multiagency and multidisciplinary partnerships. Unclear if coproduced.	Young parents	South Wales	Groups, 1-2-1	In person	Perinatal	No limit - pregnancy and beyond
Darwin et al. (2017) [57]	Doula project offering emotional support	Evaluated long-standing community-based interventions so N/A.	Vulnerable/ disadvantaged women	UK	121	In person	Perinatal	Pregnancy to 6 weeks postpartum.
Donetto et al. (2015) [42]	Health visiting outside the home and activities in children’s centres	Health visiting service designed the approaches. Not coproduced or codesigned.	Any parent	Two geographical areas in UK	Groups	In person	Postnatal	Postnatal and beyond.
Dubus (2013) [58]	Peer support intervention	The service designed the intervention. Unclear if coproduced or codesigned.	Mothers with moderate risk of depression	USA	1-2-1	In person	Postnatal	No limit in the postnatal period.
Fritzson et al. (2023) [59]	Online lullaby project with parents experiencing loneliness.	The approach was adapted from an existing intervention but not coproduced. The activity is coproducing a lullaby.	Parents who felt lonely	USA.	Group	Online	Perinatal	7 separate session- One 3.5 hours of lullaby creation sessions, and three group lullaby sharing sessions for 30 minutes.
Gale et al. (2018) [22]	Pregnancy Outreach Workers (POWs) offering support to women	A voluntary sector organisation. Unclear if coproduced or codesigned.	Women with medically high-risk pregnancies	UK	1-2-1 Group	In person	Perinatal	Pregnancy till 6 weeks postpartum.
Glavin et al. (2017) [60]	Universal perinatal support groups	Evaluated longstanding public health intervention so N/A.	Parents post-discharge from maternity hospital	Eastern Norway	Groups	In person	Postnatal	Three to four group meetings two weeks after discharge date.
Hjalmhult et al. (2014) [61]	Universal perinatal support groups	Evaluated pre-existing public health intervention so N/A.	Parents	Norway	Groups	In person	Postnatal	Three to four group meetings two weeks after discharge date.
Horton et al. (2023) [62]	Latin Dance Group intervention	Unclear who designed the intervention or if it was coproduced.	Postpartum Mothers	USA	Group	Online	Postnatal	One day session.
Ikeda et al. (2022) [63]	Public health advertisements on Instagram	The research team co-developed the adverts for the campaign with professionals.	Mothers	Japan	Self-help	Online	Post	Ads were posted for two months.
Jerksy et al. (2016) [64]	An urban art‐based community health program	Created by a service with input from multidisciplinary professionals.	Aboriginal parents	Australia	Groups	In person	Postnatal	Ongoing programme of activities.
Jiang, et al. (2022) [65]	Birth-clubs - Online peer support community	Evaluated pre-existing community-based interventions so N/A.	New mothers	China	Forum	Online	Perinatal	Duration of pregnancy.
Jin et al. (2020) [66]	Intervention for Chinese women living in Japan to overcome cultural stressors	Research team designed the intervention, and it was not coproduced.	Chinese mothers living in Japan	Japan	Self-help Groups	Messaging/phone and in person	Perinatal	Support given in the third trimester to one month postpartum.
Lesser et al. (2023) [44]	Postpartum group exercise program	Exercise class created by private company. Unclear if intervention coproduced.	Mothers	New Zealand.	Yes	In person	Postnatal	4 sessions delivered bi-weekly.
McLardie-Hore et al. (2020) [67]	Breastfeeding peer support service	Unclear who designed the intervention or if it was coproduced.	Breastfeeding mothers	Australia	1-2-1	Phone and text	Postnatal	Unlimited support but median support 5.5 months. Support was flexible.
McCarthy Quinn et al. (2019) [68]	Breastfeeding support groups	Evaluated pre-existing community-based interventions so N/A.	Breastfeeding mothers	Ireland.	Group	In person	Postnatal	Weekly groups whilst breastfeeding.
McLeish et al. (2015) [69]	Peer support	Evaluated pre-existing community-based interventions so N/A.	Mothers with disadvantages/vulnerabilities	England	1-2-1 and groups	Mixed	Perinatal	6 weeks to 2 years across multiple projects
Mcleish et al. (2016) [70]	Explore peer support for pregnant women with HIV	Evaluated pre-existing community-based interventions so N/A.	HIV positive mothers	England	1-2-1	In person	Antenatal	Duration of pregnancy and early motherhood.
McLeish et al. (2017) [71]	Organised peer support	Evaluated pre-existing community-based interventions so N/A.	Disadvantaged mothers	UK	1-2-1 and groups	Mixed	Perinatal	6 weeks to 2 years across multiple projects.
Miles et al. (2023) [72]	Online Mellow Bumps programme, an intervention to improve maternal well-being	Adapted longstanding intervention for online use. It was piloted. Unclear if codesigned or coproduced.	Pregnant disadvantaged women	Turkey	Group	Online	Antenatal	7 weekly sessions of 90 minutes.
Min-Lee et al. (2023) [73]	Doulas for migrant Australian women	Evaluated pre-existing community-based interventions so N/A.	Migrant women	Australia	Group	In person	Perinatal	Support during pregnancy.
Mkandawire-Valhmu et al. (2018) [74]	A peer support intervention for pregnant African American women	Evaluated pre-existing community-based interventions so N/A.	African American mothers	USA	1-2-1 Drop in Monthly groups	In person	Perinatal	Ongoing support for parents and families.
Parry et al. (2019) [75]	Fathers only antenatal programme	Developed by a health organisation with research evidence but unclear if coproduced.	Fathers	Australia	Group	In person	Antenatal	During the antenatal period.
Perkins et al. (2018) [43]	Community group singing intervention	It is unclear who designed the intervention.	Mothers at high risk of postnatal depression or who have postnatal depression	England	Group	In person	Postnatal	Weekly sessions for 10 weeks.
Perkins et al. (2023) [76]	Community group songwriting intervention	Developed by research team through public involvement with mothers.	Mothers experiencing loneliness or postnatal depression	England	Group	Online	Postnatal	Weekly sessions for 6 weeks.
Peters et al. (2013) [77]	Professionally-facilitated group for parents and children aged 0–4 years	Evaluated pre-existing community-based interventions so N/A.	Mothers attending Children’s Centres	England	Group	In person	Postnatal	Weekly sessions.
Rice et al. (2022) [78]	Peer support	Evaluated pre-existing community-based interventions so N/A.	Mothers.	England and South Wales.	1-2-1 Groups	Mixed	Mixed	Varied across services in the study.
Sachs et al. (2022) [79]	School-based nature social intervention for pregnant and parenting teens	The intervention was co-created with professionals but not students.	Pregnant and parenting teens	USA	Groups	Online In person	Perinatal	8 weeks and 11 sessions.
Seymour et al. (2021) [80]	Working Out Dads (WOD) intervention to support Dads with physical activity and social connections	Developed by child and family health service. Unclear if coproduced.	Fathers	Australia	Group	In person	Postnatal	Weekly for 6 weeks.
Shorey et al. (2019) [81]	Technology-based peer support	Intervention design was not discussed.	Mothers at risk of depression	Singapore	1-2-1	Technology-based – phone/emails/WhatsApp	Postnatal	At least once a week for 4 weeks
Silva-Jose et al. (2022) [82]	Online physical activity classes for pregnant women	It was unclear who designed the intervention or if it was coproduced.	Pregnant women	Spain	Group	Online	Antenatal	3 times a week during pregnancy between 8-39 weeks.
Steen et al. (2015) [83]	Community preventive mental health programme for pregnant women	A voluntary sector provider developed the programme, and it is unclear if it was coproduced.	Pregnant women and new mothers	UK	1-2-1 Group	In person	Antenatal	Structured programme of activities for 6 months.
Strange et al. (2014) [84]	Playgroups with children aged 0–5 years	Evaluated pre-existing community-based interventions so N/A.	Parents of 0-5’s –mothers focus of research	Australia	Groups	In person	Postnatal	Playgroups are usually weekly.
Strange et al. (2019) [85]	Young parents support programme	The parents codesigned elements of the young person’s programme activities.	Young parents with children younger than a year	Australia	Group	In person	Postnatal	Weekly groups.
Styles et al. (2018) [86]	Antenatal yoga-based intervention for young parents	A pre-existing intervention was adapted with a young person’s midwife.	Young parents	Australia	Group	In person	Antenatal	Duration of pregnancy – twice a week.
Taket et al. (2021) [87]	A brief relationship education program for first time parents	Developed by a health organisation but unclear if coproduced.	Parents-couples	Australia	Group	In person	Postnatal	Three sessions.
Tarleton et al. (2021) [88]	Evaluate Mellow Futures, an intervention to improve maternal well-being	Long-standing intervention adapted for people with learning disabilities. Unsure if coproduced but it was piloted.	Learning disabled mothers	UK	Group	In person	Perinatal	Prebirth (6 weeks) and post-birth (14 weeks) 2 hours weekly.
Wells et al. (2021) [89]	Prenatal and postnatal father groups in Sweden	Explored pre-existing community-based groups so N/A.	Fathers	Sweden	Groups	In person	Perinatal	During pregnancy and up to 1 year old. Average of 5 meetings.
Westbury et al. (2019) [90]	Pregnancy yoga classes	Intervention design not discussed.	Pregnant mothers	UK (Wales)	Groups Forum	Online	Antenatal	Weekly during pregnancy.

**Table 4:** Intervention types, mechanisms and outcomes

Author/Date	Intervention description	Type of intervention	Mechanism: Overcoming barriers to connection	Mechanism: Connection to similar others	Mechanism: Normalisation/acceptance of difficulties	Mechanism: Providing a positive tie	Mechanism: Meaningful activity for self	Study design and relevant outcome measures	Number of participants in each study	Description of relevant findings and/or outcomes reported
Carter et al. (2020) [54]	One-on-one peer support	Synthetic social support	Providing information and signposting to other services	Connecting with a peer support worker with lived experience	Sharing that it is normal to find parenting difficult	Non-judgemental reassurance and advice from empathetic listener		Qualitative feasibility study for RCT with control group. Follow up interviews with participants	20 women were randomised to control (10) and intervention (10). 9 from intervention and 6 from control were interviewed	Participants reported improved social support, reduced alienation and reduced isolation
Darwin et al. (2017) [57]	Doula service	Synthetic social support	Providing information and support including signposting to other services		Sharing that it is normal to find parenting difficult	Non-judgemental reassurance and advice from empathetic listener		A follow up survey with open questions about impacts. Retrospective interviews/focus groups with participants	137 women responded to the survey (response rate 21.7%).12 were interviewed	Participants reported reduced isolation and many views the Doulas as friends. Though this could lead to feelings of loss when the relationship ended
Dubus et al. (2014) [58]	Peer support	Synthetic social support	Providing information about child-care and feelings		Hearing others’ stories helped realise they were not alone	Non-judgemental advice from empathetic listener with lived experience		Qualitative approach- retrospective interviews/focus groups with participants and staff	29 mothers were and 20 volunteers were interviewed	Participants reported their feelings of isolation was reduced
Gale et al. (2018) [22]	Pregnancy Outreach Workers (POWs)	Synthetic social support	Providing information and support with benefits/finance/housing			Non-judgemental reassurance and advice from empathetic listener		Qualitative study nested in an RCT. Observations of POWs in practice and informal interviews with POWs	6 POWs were observed for 100 h in total, and informally interviewed during observation	POWs became an important part of mothers’ social networks; some were supported to make ties in their community. The end of support could be stressful
McLardie-Hore et al. (2020) [67]	Breastfeeding peer support service	Synthetic social support	Providing information about breastfeeding		Hearing others found breastfeeding hard helped to normalise the challenges	Non-judgemental reassurance and advice from empathetic listener		Nested study in RCT. Postal survey with open and closed questions and interviews with participants. Peer support evaluation inventory	360 mothers (72% response rate) including 261 who responded to open questions about positive aspects of support	Participants reported reduced isolation and made a connection their supporter
Mcleish et al. 2016 [70]	Peer support for pregnant women with HIV	Synthetic social support	Providing information about HIV and parenting and local services	Connected to others with HIV diagnosis	Sharing of diagnosis helped normalise challenges	Non-judgemental reassurance and advice. Peer support worker like a family member with valued lived experience		Qualitative approach- retrospective interviews/focus groups with participants	12 women who had either given (5) or received support (6)	Participants reported feeling less isolated, feeling cared for and supported
McLeish et al. (2015) [69]	Peer support	Synthetic social support	Provided with information about local support and mental health support	Connected with others with mental illness	Hearing from others helped to normalise challenges	Non-judgemental reassurance and advice. Peer support worker like a family member with valued lived experience		Qualitative approach- retrospective interviews/focus groups with volunteers and participants	42 mothers and 47 volunteers were interviewed or took part in focus groups	The intervention built a trusted relationship providing emotional support that participants viewed as akin to friendship or family
McLeish et al. (2017) [71]	Peer support	Synthetic social support	Provided information		Normalisation of challenges through sharing hard times	Valued not being judged and a space to be heard. Relationship was like a family member		Qualitative approach- retrospective interviews/focus groups with participants	47 mothers were interviewed	Participants reported experiencing feelings of social connection, being heard and valued
Mkandawire-Valhmu et al. (2018) [74]	Peer support and a physical safe community space for participant	Synthetic social support	Safe space and access to information, advocacy and support with finances and housing	Met other people with similar experiences of exclusion	Heard that others had similar challenges and helped normalise their feelings	Non-judgemental reassurance and advice. Peer support worker like a family member with valued lived experience		Ethnographic fieldwork and retrospective interviews/focus groups with participants	13 service users and 4 people who provided support were interviewed	The spaces and support helped participants to feel belonging, access support and make connections
Perkins et al. (2023) [76]	Community group songwriting intervention	Creative Health Approach	Provided an online safe space to meet others.	Connections to other parents with shared challenges who also like music.			Writing songs together is a shared creative activity.	RCT measuring changes to loneliness (UCLA) and Social Connectedness (SC-15).	78 began intervention and 62 completed the follow up.	Online songwriting intervention reduced postnatal loneliness and improved social connectedness.
Rice et al. (2022) [78]	Peer support	Synthetic social support	Safe environment in a group space and information about services/support	Met others with shared experiences of mental ill health	Heard that others had similar challenges and helped normalise their feelings	None-judgemental support and valued lived-experience of the supporter		Qualitative approach- retrospective interviews/focus groups with participants	24 mothers were interviewed (6) or took part in focus groups (18)	Participants reported feeling less lonely and isolated, and building friendships and connections
Shorey et al. (2019) [81]	Digital informal peer support	Synthetic social support	Information about postnatal period	Met others with shared experiences	Heard that others had similar challenges and helped normalise their feelings	Relationship with peer supporter		Randomised Controlled Trial (UCLA Loneliness Scale, Perceived Social Support for Parenting)	138 mothers were recruited to an intervention (69) or control (69) group. Attrition rate was 18.1%	Whilst the intervention did not prevent loneliness but buffered the effects of it during a confinement period
Min-Lee et al. (2023) [73]	Doulas for migrant Australian women	Synthetic social support	Information and practical support	Where women met each other in groups it was appreciated		Non-judgemental support		Qualitative approach- retrospective interviews/focus groups with doulas and birth providers	30 interviews with maternity care providers	Participants reported increased social connectedness but could have a detrimental effect when relationship ended
Jerksy et a l (2016) [64]	An urban art‐based community health program	Creative Health Approach	Information and education and provided a safe/fun space	Opportunities to meet other parents	Saw that other parents had experienced hardship and it was OK to ask for help		Art	Quasi-experimental study with qualitative follow up with participants	92 parents participated in the programme. Unclear how many gave feedback	Participants increased social connectedness in qualitative component of the study
Lesser et al. (2023) [44]	Postpartum group exercise program	Creative Health Approach		Opportunities to meet other mothers	Normalised challenges through sharing difficulties with other mothers		Physical activity and well-being: ‘something for me’	Qualitative longitudinal interviews (*n* = 3) with participants	17 mothers participated in T1, 16 in T2 and 12 in T3	Participants reported feeling less lonely, accessing support and belonging to a community. Some made friends
Perkins et al. (2018) [43]	Community group singing intervention	Creative Health Approach	Provided a safe space	A space to meet other mothers with postnatal depression			Singing was rewarding and helped mothers ‘feel like themselves’	RCT utilising qualitative methods–focus groups with participants following the singing groups	54 mothers participated in the study	Participants reported feeling they belonged to something, reconnected to themselves and their purpose, and making connections
Sachs et al. (2022) [79]	School-based nature intervention for pregnant and parenting teens	Creative Health Approach	A safe space to meet other young parents	A space to meet others in a group			Enjoyed the nature-based activities	Mixed methods pre-test post-test survey completed by participants. UCLA Loneliness Scale	17 participants (13 women and 4 men)	Participants reported increased belonging and connectedness in qualitative component. No change to loneliness in quantitative component
Silva-Jose et al. (2022) [82]	Online physical activity classes for pregnant women	Creative Health Approach		A digital space to meet other mothers			Enjoyed dance activities	Qualitative approach- retrospective interviews with participants	24 women	Participants reported feeling connected to and bonding with others
Steen et al. (2015) [83]	A programme for pregnant women involving well-being activities, building social networks and developing coping strategies	Creative Health Approach	Provided counselling	A space to meet other mothers	Opportunities to hear from others and normalise difficulties	Opportunities for peer support	Emphasised creative health approaches	Pre-test post-test survey for participants measuring well-being and resilience, including social connections element	108 mothers completed pre-test post-test survey. Response rate 56.8%	Improved scores across the measure at follow up which may indicate improved social connections
Styles et al. (2019) [86]	Antenatal yoga based intervention for young parents	Creative Health Approach	Transport costs and free activity	A space to meet other young parents			Enjoyed the yoga	Mixed-methods. Pre-and-post session evaluations then follow up interviews	30 women in the study with 16 participating in the intervention	Participants reported making social connections and friendships. The evaluations showed women felt accepted and comfortable with other group members following the yoga class
Westbury et al. (2019) [90]	Pregnancy yoga classes	Creative Health Approach		A space to meet other parents			Enjoyed the yoga	Follow-up survey including qualitative questions with participants	52 women completed the survey (response rate 41.6%)	Participants reported meeting others, building friendships and receiving support
Anolak et al. (2023) [46]	Antenatal music drawing and narrative intervention	Creative Health Approach	Encouraged to share feelings to increase connection	Meeting other with mental illness			Enjoyed drawing	Qualitative approach- follow up interviews/focus groups with participants	12 mothers	Participants reported making connections with others
Horton et al. (2023) [62]	Online Latin Dance Group intervention	Creative Health Approach	Opportunity for counselling and to discuss barriers to connection	Meet with other parents in a digital space	Normalised challenges through sharing similar experiences		Dance helped parents to connect to culture, selves and others	Qualitative approach- follow up interviews/focus groups with participants	4 mothers	Participants reported connection to baby, others, self and music
Fritzson et al. (2023) [59]	Online lullaby project with parents experiencing loneliness	Creative Health Approach	Online opportunity for those who cannot get out with children	Community-belonging through meeting shared-goals			Enjoyed participating in music and belonging to a community	Pre-test, post-test quantitative survey measuring outcomes (UCLA Loneliness Scale and Belonging scale) and asking open questions about connectedness	40 participants (30 mothers and 10 fathers)	Significant improvement in self-reported loneliness and sense of belonging
Parry et al. (2018) [75]	Fathers only antenatal programme	Psychoeducation with shared identity social support group		Connection to other fathers in groups	Sharing difficulties with other Dads normalises their feelings			Qualitative approach- follow up interviews/focus groups with participants	16 fathers and 6 staff participated in interviews or focus groups	Fathers reported feeling less alone and more connected
Donetto et al. (2014) [42]	Community Centres and activities such as parent and baby/toddler groups	Parent and baby groups		Opportunity to meet other local parents	Normalised shared challenges of parenting through group discussions			Qualitative approach- retrospective interviews/focus groups	44 mothers	Participants reported connecting to other parents and making friendships
Peters et al. (2013) [77]	Professionally-facilitated mother and child group	Parent and baby groups		Opportunity to meet other local parents				Ethnographic research utilising participant observation and in-depth interviews	12 mothers attended the groups and were observed 7 in-depth interviews	Some participants appreciated social contact, but support and connections were limited by feelings of judgement and professional facilitation
Strange et al. (2015) [84]	Informal Playgroups with children aged 0–5 years	Parent and baby groups		Opportunity to meet other local parents	Normalise shared challenges of parenting including breastfeeding			Qualitative approach- interviews/focus groups with participants	39 mothers from 16 mothers’ groups and 13 playgroups	Participants reported making social connections, building friendships and feeling connected to the community
Augustin et al. (2023) [48]	Online psychoeducation materials with online support group with early childhood crying, sleeping, and feeding problems	Shared identity social support group		An online forum to connect with others with similar challenges	Education on feeding and crying issues and a forum to share difficulties with others and therefore normalise their experiences			Controlled Trial – surveys with measures perceived social support and a measure of social isolation	136 participants (Intervention group (73) and Waitlist group (63))	Reduced social isolation at follow up; no evidence of changed social support
Berg et al. (2018)[49]	Web-based support, including peer support, on women with Type 1 Diabetes	Shared identity social support group		Digital space for parents with Type 1 to connect	Education on Type 1 diabetes challenges helped normalise difficulties and a forum to share with others			Case-study of the design of online support	N/A observation of online forums	The study found people utilised online peer support but used it much less if facilitation stopped
Bess et al. (2014) [50]	Place-based parent education initiative	Shared identity social support group	A safe place for parents and a creche, transport and free meals for participants	Opportunities for local parents to meet		Positive relationships with programme staff	Fun group activities offered to parents including lunch and trips	Qualitative approach- social network analysis and interviews with participants	69 participants	Participants reported expanded social networks and positive relationships with staff
Birtwell et al. (2015) [51]	Explore womens’ experiences of Mellow Bumps intervention	Shared identity social support group	Counselling and therapeutic work. Free transport and creche provided	Meeting other parents in groups and discussing their history and challenges	Women reported they were not alone with difficult childhood experiences			Qualitative longitudinal interviews with participants	8 participants	Some participants reported making friendships and many received support
Brookes et al. (2015) [52]	Antenatal classes- Baby Steps programme for minority ethnic parents	Shared identity social support group	Exploring local culture, providing information, and a creche facility	Meeting other minority ethnic parents in groups		Positive relationship with staff members who provided support		Qualitative interviews with participants	14 participants (3 fathers and 11 mothers)	Participants reported increased support from professionals and peers
Buston et al. (2018) [41]	Mellow Bumps intervention	Shared identity social support group	Counselling and therapeutic work	Meet other expecting parents in groups who had experienced similar challenges				Process evaluation of Mellow Bumps including interviews and evaluation forms	16 mothers and 5 facilitators interviewed. 115 evaluation forms from participants and 43 from facilitators	Participants reported to feel less isolated and less alone
Buultjens et al. (2018) [53]	Antenatal 3rd trimester psychoeducational group	Shared identity social support group	Provides therapeutic advice and information about being parent	Meet other expecting parents in groups	Topics help to share and normalise challenges experienced in early parenthood			Controlled trial exploring outcomes for participants. Measures of social support	18 women (10 intervention and 8 control) took part	Positive increase in social support but inconclusive due to sample size
Chatwin et al. (2021) [55]	Facemums – an online midwife facilitated virtual community	Shared identity social support group		Connecting with other mothers online in digital forums		Connection to the midwife facilitator		Online survey with closed and open questions sent to participants	156 participants (response rate 49%), including 105 that completed open ended questions	Participants reported reduced feelings of isolation in the open text questions
Glavin et al. (2017) [60]	Well Child Clinics maternity group	Shared identity social support group		Regular groups to meet other new parents	Normalising challenges of parenthood through discussion with other parents			Qualitative approach – focus groups with participants	30 mothers participated	Participants reported new social networks, making friends and exchanging support
Hjalmhult et al. (2014) [61]	An exploration of parents’ perspectives of Well-child clinics	Shared identity social support group		Regular groups to meet other new parents	Normalising challenges of parenthood through discussion with other parents			Qualitative approach- interviews/focus groups with participants	18 mothers and 3 fathers participated	Parents wanted to make social connections through these groups. The study explored some facilitators and barriers
Jiang et al. (2022) [65]	Birth-clubs—Online peer support community	Shared identity social support group		Digital space to connect with other parents with children of similar ages				Survey of participants in online support groups measuring changes to social support, with open questions	500 mothers	Participants reported that online birth clubs provided social support which was similar to support offered by family and friends
Jin et al. (2020) [66]	Intervention for Chinese women living in Japan to overcome cultural stressors	Shared identity social support group	Promotes cultural understanding	Provides an online social network				Pre-test, post-test survey measuring social support and qualitative interviews with participants	18 participants (10 intervention and 8 control)	No impact on long term social support May support cultural integration
McCarthy Quinn (2019) [68]	Breastfeeding support groups	Shared identity social support group		Meeting other parents who breastfeed	Normalising challenges and experiences of breastfeeding			Qualitative study nested in an RCT. Interviews with participants	15 mothers	Participants reported feeling part of a community, making connections and building friendships
Miles et al. (2023) [72]	Online Mellow Bumps	Shared identity social support group	Access to therapy and provide service for those who can’t attend in person	Online meetings for similar others in groups				Pre-test post-test survey collected through routine evaluation measuring maternal social connectivity	128 mothers	Improved social connectivity at follow up
Seymour et al. (2021) [80]	Working Out Dads (WOD) intervention	Shared identity social support group		Connection to other fathers in groups			Group-based exercise class	Qualitative approach- follow up interviews/ focus groups with participants	11 fathers	Participants reported making social connections
Strange et al. (2018) [85]	Young parents support programme including peer support, groups and professional support	Shared identity social support group	Offering personal and holistic care to overcome challenges	Participating in group discussions with other young parents		Staff offer non-judgemental and reassuring advice		Qualitative interviews with young parents and a focus group with facilitators	20 parents (19 mothers and 1 father) and 5 facilitators took part	Parents reported developing friendships, support networks and links with community services
Taket et al. (2020) [87]	A brief relationship education program for first time parents	Shared identity social support group	Psychological support and communication skills	Meeting other parent-couples	Normalisation of difficulties through sharing in groups of peers			Routinely collected surveys were analysed and interviews with facilitators and participants	40 parents (fathers (14) and mothers (26). Interviewed. 342 parents completed surveys	Participants reported the social interaction within the group as being an outcome
Tarleton et al. (2020) [88]	Mellow Futures, an intervention to improve maternal well-being	Shared identity social support group	X childcare lunch	Connections to similar others in groups				Qualitative longitudinal interviews with participants	36 mothers	Participants reported building connections, developing social skills, reduced social isolation and obtaining emotional support
Wells et al. (2020) [89]	Prenatal and postnatal father groups in Sweden- only open for fathers	Shared identity social support group		Meeting other parents and fathers in groups				Online survey completed by participants. Closed questions about impact on loneliness and social networks	67 fathers with a response rate of 77%	Participants reported reduced loneliness and improved social networks
Aube et al. (2019) [47]	Wraparound holistic support delivered to migrant mothers in a community centre	Holistic, place-based and multidisciplinary	Providing a physical place to feel safe	Meeting other migrants/refugees		Staff offer support		Ethnographic study utilising observation and depth interviews	24 mothers participated (9 interviewed, 17 observed)	Participants reported building positive and supportive relationship and belonging to a community
Darra et al. (2020) [56]	Multi-agency project to support young parents	Holistic, place-based and multidisciplinary	Offering personal and holistic care to overcome challenges	Meeting other young parents and attending groups		Staff are non-judgemental		Participant observation and focus groups with participants	18 participants (16 women and 2 men)	Participants reported building friendships and receiving support
Ikeda et al. (2022) [63]	Public health advertisementcampaign on Instagram	Awareness Campaign	Information about where to get support		Seeing that others are lonely through adverts			Pre-test post-test survey with measures of loneliness (UCLA)	419 mothers completed the pre-test post-survey (dropout rate 15%)	Mothers’ feelings of loneliness decreased after reading the online messages, particularly for women with financial instability

Many of the papers studied the outcomes and processes of interventions or approaches that were already widely used, such as peer support groups or longstanding local services. Many others were novel interventions designed by research teams. In some studies, it was unclear who had designed the intervention or if it was co-produced. Three studies reported that interventions had been designed by research teams with input from professionals [63, 64, 79]. Only one paper described in depth a process of public involvement to design an intervention [76]. Three papers explored interventions that were being piloted [55, 72, 88].

Most studies utilised retrospective methods to explore intervention outcomes (*n* = 27), including qualitative methods (*n* = 22) [42, 46, 50, 52, 57, 58, 60–62, 67, 69–71, 73, 75, 78, 80, 82, 84–86, 88], surveys (*n* = 4) [55, 65, 89, 90] and evaluation methods (*n* = 2) [41, 87]. Four studies utilised ethnographic methods to explore in-depth processes of community-wide services [47, 56, 74, 77]. One study was a case study exploring intervention process [49]. One study was a qualitative feasibility study for a Randomised Controlled Trial (RCT) using before and after focus groups [54]. Fourteen studies utilised experimental methods to explore changes in outcomes over time. Nine studies collected longitudinal data to explore the impact of an intervention. These included two that used qualitative methods [44, 51] and eight that used pre-test, post-test survey data [59, 63, 64, 66, 72, 79, 83]. Three studies were RCTs [43, 76, 81], including two measuring changes to loneliness or social support [76, 81] and one using qualitative approaches to track outcomes [43]. Two were controlled trials [48, 53].

Six studies measured changes to loneliness using an outcome measure [59, 63, 76, 79, 81, 89] and four studies explored interventions where participants reported reduced perinatal loneliness in the qualitative findings [44, 74, 75, 89]. The other studies reported proximate determinants of loneliness, which included increased social support (*n* = 20), reduced social isolation (*n* = 21), new friendships (*n* = 11), new supportive relationships (*n* = 3), social connections (*n* = 11), increased social networks (*n* = 11), improved feelings of belonging and/or identity (*n* = 5) or increased social capital (*n* = 2).

Few studies collected comprehensive demographic data (*n* = 10) and some did not collect any (*n* = 6). Most interventions were aimed at mothers, with only three specifically for fathers [75, 80, 89]. Eleven of the interventions were aimed at all parents, though two of these explored the impacts for mothers only [60, 85]. When interventions were aimed at both parents, participation by fathers ranged from 5% to 35%. No studies examined interventions specifically aimed at LGBTQ+ parents and only one collected data on sexuality [59].

Some interventions specifically targeted populations considered or known to be at a greater risk for loneliness and/or social isolation. For example, interventions for parents of babies with feeding and crying issues [48], mothers with Type 1 diabetes [49], parents experiencing loneliness [59, 76], at risk of or with postnatal depression [43, 58, 76, 81], migrant mothers [47, 52, 66, 73, 74], parents with learning disabilities [88], vulnerable parents [22, 41, 50–52, 54, 57, 64, 69, 74, 78, 79] first-time parents [53, 87], breastfeeding mothers [67, 68], medically high-risk pregnancies [46], and young parents [56, 64, 79, 85, 86].

When interventions specifically targeted low income or ethnic minority populations [47, 50, 52, 56], representation of these communities was high, suggesting that tailored interventions successfully engaged these groups. Only one study recorded information about religion [58].

Interventions were delivered in a range of ways (see Table 3) with most either in-person or online group-based activities (*n* = 31). There were 11 delivered in one-to-one sessions. Two were delivered one-to-one via phone or text. There were 12 online group-based interventions, and all of these were delivered post-2020, which suggests that Covid-19 social distancing policies (introduced in March 2020) might be responsible for this rise in online support. Half of the interventions delivered online offered at least one group session and six featured a community forum. Five interventions involved accessing self-help resources and two utilised a physical drop-in space in community centres.

#### Types of intervention

The interventions were categorised into six ‘types’ following analysis of the support they delivered (see Table 4). Types included 1) synthetic social support, 2) shared-identity social support groups, 3) parent and baby groups, 4) creative health approaches, 5) holistic, place-based and multidisciplinary support, and 6) awareness campaigns. Some of the interventions could fit into more than one ‘type’; when this was the case, we used the type that most accurately described the intervention.

##### Synthetic social support

Gale et al. [22] describes synthetic social support as support provided by a professional or volunteer in a service through a non-reciprocal and time-limited relationship. Support includes offering direct emotional, practical advice or information, and/or help to connect with other services and build social networks. We identified 12 papers describing synthetic social support interventions, including peer support [22, 54, 58, 67, 69–71, 74, 78, 81] and doula support [57, 73].

Synthetic social support was either provided through group-based peer support [69, 71], or offered in a one-to-one relationship [22, 54, 57, 58, 67, 69–71, 74]. It was provided by volunteers [54, 57, 69, 70], or paid staff [22, 58, 67, 81] and through charities [69, 78] or within a statutory service [22, 54, 67]. It was offered via the telephone [67, 81], digitally [81], or in person [22, 54, 57, 58, 69, 70, 74]. Three papers explored services that delivered a mix of the support described above [69, 71, 78].

People were referred to or accessed synthetic social support for a range of reasons including that they were at risk of or experiencing depression [54, 58, 78, 81], they were considered vulnerable or marginalised [22, 57, 69–71], or they were experiencing cultural exclusion such as racism [73, 74]. One intervention was offered to any parent to prepare for breastfeeding [67].

Some issues were identified with the time-limited non-reciprocal nature of the relationship, which left some participants experiencing distress and further isolation [22, 57, 69].

##### Shared-identity social support groups

Shared-identity social support groups include spaces where participants with similar characteristics, or facing shared challenges, come together for support [23]. This theme differed from synthetic social support because it was group-based and involved others with shared experiences. Unlike synthetic social support, it was often reciprocal and might not be time limited. There were 19 studies that described or evaluated shared-identity social support groups. Six were a stand-alone intervention [55, 60, 61, 65, 68, 89] and 13 were facilitated as part of another perinatal intervention/service [41, 48–53, 66, 72, 75, 85, 87, 88].

Most stand-alone groups were also facilitated by professionals who encouraged peers to make connections [55, 60, 61, 68, 89] and some also offered their support during groups [55]. Three were online support communities for new parents [55, 65] including one which was midwife-facilitated [55]. Two were in-person groups delivered by public health nurses that aimed to form long-lasting social connections between parents with similar due dates and postcodes [60, 61]. Two were support for groups with specific needs, including a breastfeeding support group run by the voluntary sector [68] and a group for fathers, which was delivered by a child health nurse and father volunteers [89].

There were 13 interventions that offered shared-identity social support (as above) as part of perinatal psychoeducation or therapeutic intervention designed to either increase access to perinatal support for a minoritised group [41, 50–53, 66, 72, 75, 85, 88], or to provide information to overcome specific challenges [48, 49, 87].

Two studies explored an education intervention coupled with access to an online support forum for parents experiencing a specific health or care issue. One explored support with childhood crying and feeding [48], and another explored support for pregnant women with Type 1 diabetes [49].

The other 11 interventions sought to provide antenatal or postnatal education coupled with support to overcome isolation or social challenges experienced due to their specific characteristics. These included being vulnerable [41, 51, 72], a first-time mother [53], an ethnic minority parent [52], a young parent [85], a father [75], or a Chinese woman living in Japan who felt culturally isolated [66], or having an intellectual disability, [88]. One intervention aimed to improve the relationships between partners who were first-time parents [87]. Another was a place-based parent education initiative in a disadvantaged neighbourhood that aimed to connect local parents and offered education classes, discussion groups and trips [50].

The online intervention that aimed to encourage social connection between parents with Type 1 Diabetes found that sustained facilitation was important to encourage group online interactions [49].

##### Creative health approaches

Creative health refers to opportunities for arts, creativity, culture and sport to be embedded in public health [91]. There were 13 interventions that offered opportunities to connect with others whilst also engaging with a creative activity [43, 44, 46, 59, 62, 64, 76, 79, 80, 82, 83, 86, 90]. They included an arts-based community programme [64], a nature-based intervention in a school [79], an arts-based intervention on a mental health ward [46], exercise groups [44, 80, 82], yoga groups [86, 90], a dance group [62], and singing or song and lullaby writing groups [43, 59, 76]. One programme provided a range of creative activities [83]. All interventions provided an opportunity to participate in shared activity. Some included facilitated or planned social time afterwards and/or between sessions [43, 44, 76, 80] or discussions during sessions [46, 64, 76, 79].

Sessions could be in person [43, 44, 46, 64, 80, 83, 86], online [59, 62, 76, 82, 90] or a mix of both [79]. The sessions were mostly delivered by creative health practitioners, for example, exercise class leaders, song writers or artistic workshop facilitators, but antenatal yoga was delivered by a midwife, and some activities were delivered or co-delivered by therapists [46, 64, 80].

Some interventions were offered to all parents [44, 62, 82, 83, 90], but many were designed for parents experiencing some difficulty or from populations under-served by health services. For example, some interventions were specifically designed for people who were: Aboriginal [64], young parents [79, 86], fathers [80], experiencing or at risk of perinatal mental illness [43, 46, 76], or parents who felt lonely [59, 76]. Many authors reported that creative approaches could be utilised to engage with populations who might not access more traditional services or support.

##### Holistic, place-based and multidisciplinary support

Two interventions offered a range of holistic, place-based and multidisciplinary support across the perinatal period and beyond. Both these interventions involved different professionals working with parents to address their personal challenges, which could include poverty, access to education or housing, relationship difficulties, domestic violence, or mental illness. One was multidisciplinary and offered peer support, antenatal classes, parenting classes and social opportunities for young parents aged 15–24 [56]. Another offered a multidisciplinary perinatal health and social centre providing medical and educational services, groups and a physical safe-space for migrant women [47]. These interventions were not time-limited and offered ongoing support to work with women to overcome their complex personal challenges which included developing their social networks and accessing support.

##### Parent and baby groups

Three papers explored parent and baby groups as a site of connection. These are organised community-based groups to which parents could take their children, often based in local community facilities. They provide opportunities for parents to meet other local parents with children of similar ages. Three studies explored pre-established parent and baby groups [42, 77, 84]. One explored weekly playgroups set up in the community by churches, statutory sector organisations or community groups [84]. Two explored services provided in UK Children’s Centres, including playgroups and baby classes [42, 77]. These activities were co-located with services so that parents could access support including advice and information from professionals [42, 77] and counselling [77]. All parents also had opportunities to meet other local parents and get informal peer-to-peer support.

One study highlighted that whilst some parents appreciated social contact with other parents, there was a tension between the professional agenda of a facilitated group and the needs of parents, who might feel judged [77]. Furthermore, it could be challenging to balance playing with children whilst socialising with others, which limited opportunities for connection [77].

##### Awareness campaign

There was one intervention that was a social media campaign of four adverts co-produced with public health nurses on Instagram in Japan to educate all mothers about the possibilities of experiencing loneliness in the perinatal period. The intervention aimed to reduce feelings of loneliness through supporting new mothers to realise that feeling lonely was a common experience, and to encourage them to ask for support [63]. The adverts were targeted at mothers of 4-month-olds in a Japanese city as part of a public health campaign.

### Intervention mechanisms

We identified five mechanisms common across these intervention types that might help prevent or reduce loneliness and/or its proximal determinants (see Table 4). Many interventions utilised all mechanisms and the mechanisms overlapped and were related to each other.

#### Connections to similar others

Most interventions aimed to provide opportunities for people in the perinatal period to meet others experiencing similar challenges, for example, their peers or peer supporters (Table 4). These connections helped parents to feel less isolated and lonely because they could share their experiences with others who understood. Parents realised that they were not the only ones finding their transition to parenthood challenging. Some interventions offered opportunities to meet other parents in the perinatal period more generally [42, 44, 50, 53, 60–62, 65, 77, 78, 81–84, 87, 90], but some aimed to connect parents experiencing specific challenges in addition to parenthood. For example, some interventions provided opportunities to connect with parents with shared clinical diagnoses, such as HIV [70], Type 1 diabetes [49], or perinatal mental illness [54, 69, 71, 76]. Some interventions provided opportunities to meet others facing shared social challenges, including being refugees and migrants [47, 52, 66, 73, 74], experiencing loneliness [43, 46, 59, 78], being considered vulnerable [41, 51, 72], or living with a learning disability [88]. Some groups offered support with caring for a baby such as when breastfeeding [68], or if experiencing issues with feeding or crying [48]. Others provided connections based on age (young parents) [56, 64, 79, 85, 86], and gender (being fathers) [75, 80, 89].

#### A positive ‘tie’

Many participants in the interventions did not have a network of supportive people, such as partners, family or friends, that help them overcome challenges in the perinatal period. This lack of a network resulted in participants feeling isolated and lonely. Some interventions offered parents who were objectively isolated and/or were experiencing loneliness a much-needed connection, or ‘positive tie’, who offered formal support through a professional or volunteer relationship. The synthetic social support and multidisciplinary, holistic and place-based interventions provided this through a relationship with a peer support worker or a doula. Other interventions provided this through online support delivered by a midwife [55], or general support from staff delivering interventions [47, 50, 52, 56, 85]. Participants in interventions that created a positive tie commonly valued three qualities in this relationship (see Table 4): that the person was non-judgemental, offered reassurance and was empathetic. Some also valued the positive tie having shared lived experience (see theme above, connection to similar others).

#### Normalisation and acceptance of difficulties

Many papers reported that participants felt ‘alone’ with finding parenting difficult. Parents found it hard to share their difficulties due to a perceived stigma of not meeting a cultural expectation that parenthood is a wholly positive experience. This sense of isolation was compounded for parents also experiencing stigmatising situations/conditions such as mental health difficulties, HIV, young parenthood, childhood trauma or care experiences. Participants valued a safe space to discuss, accept and normalise their challenges through discussing their experiences with others in a safe space (either individuals or in groups) (Table 4). Participants reported that hearing stories from peers, who were experiencing/had experienced similar challenges, helped them to feel less alone [58, 69–71] and could empower parents to ask for assistance [64]. One of the aims of the social media awareness campaign intervention was to help parents realise that feeling lonely was a normal experience and to ask for help [63].

#### Overcoming barriers to social connection

Many papers identified that parents experienced multifaceted barriers to social connection, including language barriers, a lack of information about local support and services, being on lower incomes and not being able to afford transport or activities, distrusting services, a lack of confidence, poor relationship skills, or having different cultural preferences. Interventions often worked with participants to overcome their personal barriers and thus helped to facilitate social connections. Some offered information about local services and support [22, 41, 47, 57, 64, 67, 69, 71, 73, 85]. One offered a physical safe space for migrant and refugee communities who felt culturally excluded [47]. Others provided advocacy, or language support, or offered to facilitate access to further support [57, 69, 78]. Some removed financial barriers for attending groups, covered transport costs, or offered a free creche [41, 51, 72, 86, 90]. Some offered psychological support to overcome relationship or communication difficulties, including counselling [62], or group-therapy [41, 46, 51, 72]. Others offered spaces to reflect on the impacts of childhood trauma that helped with normalising their difficulties [41].

The creative health interventions offered an activity alongside gaining support that encouraged attendance and removed a barrier for people less likely to access formal health and care settings. Holistic, place-based and multidisciplinary support specifically worked with parents to overcome all the barriers to connection in their life [47, 56]. The interventions delivered online also provided parents with the opportunity to access support and participate in social activities whilst at home. Whilst this was essential during the Covid-19 pandemic, parents can also struggle to leave the house due to their caring responsibilities. Online interventions removed this barrier to making connections.

#### Offering meaningful activity

Parents who feel lonely often report that they feel distanced from their sense of self [7] and may not have time to do things they used to enjoy such as their hobbies [18]. We argue that this finding could lead to feelings of both existential and social loneliness if parents lose their sense of identity and/or do not have time for their usual activities. Participants in interventions that utilised creative health approaches valued the opportunity to engage in an activity that they enjoyed themselves, which supported their health and/or well-being. For example, parents enjoyed opportunities to take part in activities with their babies whilst also participating in exercise [44, 80, 82], mindfulness and yoga [86, 90], art [46], singing or songwriting [43, 59, 76], or being outside in nature [79].

## Discussion

The review aimed to identify interventions that could reduce perinatal loneliness. Similar to previous reviews of loneliness interventions [7, 23], we found that broadening searches to include proximate determinants of loneliness, such as social connectedness, was a useful strategy to include a wider range of studies. Our review was timely because just over two thirds (42%) of papers had been published after the cut off for previous reviews (2020), indicating a rapid increase in evidence in this specific area in the last four years. It was therefore useful to conduct this review post-pandemic and in a climate of increasing interest in both loneliness generally, and perinatal loneliness specifically.

Similar to previous reviews synthesising studies relating to perinatal loneliness [3] or parental loneliness [4], our review highlights that very few interventions specifically focussed on reducing perinatal loneliness. Only six studies measured changes to loneliness using a quantitative outcome measure and these were all published post-2021 [59, 63, 76, 79, 81, 89]. This perhaps indicates growing awareness of the need to address perinatal loneliness. A further four papers described interventions which identified reduced loneliness as an outcome in a qualitative theme or finding [44, 63, 74, 75]. There were no interventions that explored existential loneliness, with few describing emotional or social loneliness.

The review developed a categorisation of six intervention types that might have impact on loneliness and proximal determinants. These include 1) synthetic social support, 2) shared identity social support groups, 3) creative health approaches, 4) parent and baby groups, holistic, 5) holistic, place-based, and multidisciplinary support, and 6) awareness campaigns. Some of these intervention types, such as shared-identity social support groups and peer support, are similar to interventions for loneliness in other populations [23]. However, common and effective approaches used to reduce loneliness in other populations, such as befriending and volunteering [28, 92, 93], are noticeably absent from the perinatal intervention literature. Parent and baby may be seen as an inseparable dyad and volunteering and befriending may therefore not seem appropriate, although they could be.

Most of the interventions explored in this review were delivered face-to-face. However, similar to the expansion of digital interventions to reduce loneliness for older people [94], the Covid-19 pandemic appears to have influenced the way interventions were delivered for the perinatal population. For example, the 12 online interventions reported in this study were delivered post-2020. Digital technology including information and advice Apps, sessions delivered over videoconferencing, and support through online forums were welcomed by participants in the interventions. Creative activities, such as singing and songwriting [43, 59, 76], dance [62] and exercise [82], could also be delivered online through videoconferencing. The review shows that digital and online interventions may be promising for new parents and could remove barriers for participation, such as not being able to attend activities in the evening due to having no child-care. However, findings from studies exploring online interventions also highlighted challenges including the need for sustained facilitation [49]. The potential of online interventions to build sustained social networks in the perinatal period compared to face-to-face should be evaluated.

The review presents a novel contribution to our understanding of mechanisms for interventions that may reduce perinatal loneliness. The potential mechanisms identified during data extraction were 1) providing an opportunity for social connection, 2) providing a positive tie, 3) normalising and accepting challenges, 4) support to overcome barriers to social connection, and 5) providing a meaningful activity. This framework is a useful starting point for future research which could explore, interrogate, and refine these mechanisms, establish the relationship between them, and understand their relationship with different forms of loneliness (emotional, social and existential). Each mechanism may have differing impacts on different forms of loneliness. For example, providing a positive tie may overcome emotional loneliness, whereas providing a meaningful activity and normalising challenges in parenthood may overcome existential loneliness. More research is needed to explore mechanisms, perhaps using realist methods [95] to understand what works about specific interventions with specific demographic groups to address loneliness.

Studies were included in this review if the interventions were reported to impact positively on social outcomes (Table 4). Many of the interventions were not designed with the primary purpose of improving social outcomes. The majority of studies utilised qualitative exploratory methods to explore outcomes of interventions for participants. Whilst 12 studies utilised experimental designs (RCT, controlled trials or pre-test post-test surveys with no control) many of these identified social outcomes through open-text qualitative components on surveys or follow up interviews rather than utilising pre-test post-test measures (Table 4). Future research studies should use robust experimental research designs to evaluate outcomes, including for different demographic groups such as LGBTQ+ parents.

It was notable that very few interventions in this review were co-designed or co-produced (Table 3). Interventions for perinatal loneliness and proximate determinants should be developed with a strong theoretical underpinning and rationale and with input from the people who will use the services. There is value in co-producing interventions so that they are responsive to local and individual needs; the UK Government’s Best Start for Life Policy recommends that all local support is co-designed [96]. Future research could co-produce and design perinatal loneliness interventions and ensure their effectiveness is formally evaluated.

The geographic spread of interventions that may reduce perinatal loneliness identified across countries in the global north supports previous research that has highlighted loneliness for this population is a trans-cultural and trans-global issue [3, 4, 6]. However, the searches for this review did not identify papers exploring interventions in the global south, which could be because loneliness is less prevalent or prioritised, or that that the searches were not inclusive enough, for example, by only including studies published in English. The review identified some interesting differences in the types of support offered to new parents in the global north. For example, Scandinavian countries and Australia had adopted a universal public health approach in which professionals facilitated free postnatal social support groups with the distinct aim of creating social connections for new parents. The Scandinavian approach was also father inclusive because fathers could attend the postnatal groups. The UK offered Health Visiting Services for all parents, whereas in the USA, health visiting was only available for disadvantaged and marginalised women. There were also interesting programmes for migrant women from collectivist cultures; for example, women moving from China to Korea or Japan to adjust to a more individualistic culture with less postnatal support. Future research could compare the types of parental support to reduce loneliness in different countries, cultures and social policy regimes.

Many papers described or evaluated interventions aimed at groups known to be at risk of perinatal loneliness, including refugee and migrant populations, young mothers, and disadvantaged and vulnerable groups [5]. However, there were many groups missing from the intervention literature. Only three studies specifically explored an intervention for fathers, and none specifically for LGBTQ+ groups, despite evidence suggesting loneliness is prevalent in LGBTQ+ parents in the perinatal period [3]. Indeed, only one study actively collected data on sexuality [59], thus rendering the presence and experiences of LGBTQ+ parents largely invisible. Other notable absences were interventions for neurodiverse populations, parents with chronic poor health conditions or a disability, or interventions for parents of children with these experiences. Only one study explored an intervention for parents with a learning disability. Few studies recorded participants’ ethnicity or religion which is important when considering health inequalities. This review has highlighted that more research is needed to explore specific interventions for loneliness, or adaptations of existing interventions to meet the needs of different populations.

### Limitations and further research

The review fulfilled its aims of identifying many promising approaches and mechanisms to reduce perinatal loneliness. However, there are limitations. Our aim to explore a wide range of approaches through a restricted scoping review approach meant that we did not quality appraise the studies or exclude studies on criteria relating to methodological quality [33, 34]. Consequently, the summaries of intervention outcomes were descriptive, and did not discuss issues including risk of bias, affect sizes or reliability.

We also did not synthesise evidence on intervention effectiveness. We therefore cannot compare interventions to make inferences about which are more effective and for whom. A valuable contribution to the evidence in future would be a review that investigated intervention effectiveness by exploring rigorous experimental studies that included validated measures of loneliness (and proximate determinants).

Our restricted review approach meant one reviewer selected studies and extracted data, which could lead to increased errors, although a sampled 10% of the selected papers were checked by a second person. Excluding articles in the grey literature and articles published before 2013 might mean we missed important results and data. Excluding non-English articles also limited the review’s scope.

## Conclusions

The review identified and synthesised approaches that could address perinatal loneliness and its proximate determinants. There has been an increase in published research studies specifically focussed on interventions for perinatal loneliness since 2020, suggesting an increasing awareness of the issue. The broad search criteria identified six types of intervention and five intervention mechanisms that may support both intervention design and evaluation in the future. Online and creative approaches to perinatal well-being have also become more common since 2020. The review identified gaps in the research, including that few interventions were developed to overcome different forms of loneliness, such as emotional or social loneliness, and none for existential loneliness, which may be common in early parenthood. Further research is needed to identify and review papers exploring interventions in the global south; review intervention effectiveness, including for different perinatal sub-populations; and co-produce and evaluate interventions, including for under-served groups such as fathers, LGBTQ+ communities, and cultural and religious minorities. The review also identified that digital approaches are becoming more common, and more research is needed to explore their effectiveness compared to face-to-face approaches.

## Data Availability

No datasets were generated or analysed during the current study.
